# Oligocene-Miocene Mammalian Fossils from Hongyazi Basin and Its Bearing on Tectonics of Danghe Nanshan in Northern Tibetan Plateau

**DOI:** 10.1371/journal.pone.0082816

**Published:** 2013-12-23

**Authors:** Qiang Li, Xiaoming Wang, Guangpu Xie, An Yin

**Affiliations:** 1 Key Laboratory of Vertebrate Evolution and Human Origins of Chinese Academy of Sciences, Institute of Vertebrate Paleontology and Paleoanthropology, Chinese Academy of Sciences, Beijing, China; 2 Department of Vertebrate Paleontology, Natural History Museum of Los Angeles County, 900 Exposition Blvd., Los Angeles, California, United States of America; 3 Gansu Provincial Museum, Lanzhou, Gansu Province, China; 4 Department of Earth and Space Sciences and Institute of Geophysics and Planetary Physics, University of California Los Angeles, Los Angeles, California, United States of America; University of Pennsylvania, United States of America

## Abstract

A shortage of Cenozoic vertebrate fossils in the Tibetan Plateau has been an obstacle in our understanding of biological evolution in response to changes in tectonism, topography, and environment. This is especially true for Paleogene records, so far known by only two sites along the northern rim of the Plateau. We report a Hongyazi Basin in northern Tibetan Plateau that produces at least three mammalian faunas that span Oligocene through late Miocene. Located at the foothills of the Danghe Nanshan and presently connected to the northern margin of the Suganhu Basin through the Greater Haltang River, the intermountain basin is controlled by the tectonics of the Danghe Nanshan to the north and Chahan’ebotu Mountain to the south, making the basin sediments well suited for inferring the evolutionary history of these two mountain ranges. At the bottom of the local section, the Oligocene Haltang Fauna is best compared to the early Oligocene *Desmatolagus*-*Karakoromys decessus* assemblage in the Dingdanggou Fauna in Tabenbuluk Basin. The Middle Miocene Ebotu Fauna from the middle Hongyazi section shares many taxa with the late Middle Miocene Tunggur mammal assemblage in Inner Mongolia, such as *Heterosminthus orientalis*, *Megacricetodon sinensis*, *Democricetodon lindsayi*, and *Alloptox gobiensis*. Toward the top of the section, the Hongyazi Fauna includes late Miocene elements typical of *Hipparion* faunas of North China. All three faunas are of typical North China-Central Asian characteristics, suggesting a lack of geographic barriers for faunal differentiation through the late Miocene. Sedimentary packages producing these faunas are arrayed from north to south in progressively younger strata, consistent with a compressive regime to accommodate shortening between Danghe Nanshan and Chahan’ebotu Mountain by thrust faults and folds. With additional constraints from vertebrate fossils along the northern flanks of the Danghe Nanshan, an eastward propagation of the Danghe Nanshan is postulated.

## Introduction

Cenozoic vertebrate fossils within the Tibetan Plateau have been generally scarce, often for lack of appropriate deposits or difficulties in access to exposures, but also for limited paleontological efforts devoted to the plateau exploration [1]. This shortage of fossil-producing sites has been a major obstacle in our understanding of biotic response to dramatic changes in orogeny, topography, climate, and environment. Known fossil sites range from early Miocene to Pliocene [2], [3], [4], [5], [6], [7], [8] and knowledge about early Cenozoic (Oligocene and earlier) has so far remained elusive, except along the northern margins of the plateau [9], [10]. Our discovery of a fossiliferous Hongyazi Basin in northern Tibetan Plateau, previously known by two late Miocene fossil sites [11], [12], but now expanded to include a series of at least three mammalian faunas, spanning from early Oligocene through late Miocene, represents a breakthrough with important consequences.

The Hongyazi Basin is at the southern foothills of the Danghe Nanshan along the northern margin of the Tibetan Plateau. Presently connected to the northern margin of the Suganhu Basin, which is adjacent to Qaidam Basin to the west, through the Greater Haltang River, this intermountain basin is controlled by the tectonics of the Danghe Nanshan to the northeast and Chahan’ebotu Mountain to the southwest. This makes the basin sediments well suited for inferring the evolutionary history of these two mountain ranges.

Cenozoic sediments in Hongyazi Basin are fragmented and juxtaposed by a series of thrust faults. Three partial sections are exposed and vertebrate fossils were collected from each to help constrain the basin chronology. Faunas with age-diagnostic taxa from these sections help reconstruct basin history as well as infer zoogeographic relationships with faunas elsewhere, particularly those along the outer margins of the Tibetan Plateau. The following is a report on the new vertebrate faunas.

## Materials and Methods

All necessary permits were obtained for the described study, which complied with all relevant regulations. Field permits in the Hongyazi area were granted by the county bureau of Ministry of Land and Resources in the government of Aksai County, Gansu Province. All vertebrate fossil specimens collected belong to and are housed and catalogued in the Institute of Vertebrate Paleontology and Paleoanthropology (IVPP), Chinese Academy of Sciences, in Beijing.

### Geologic Setting

**Figure 1:**
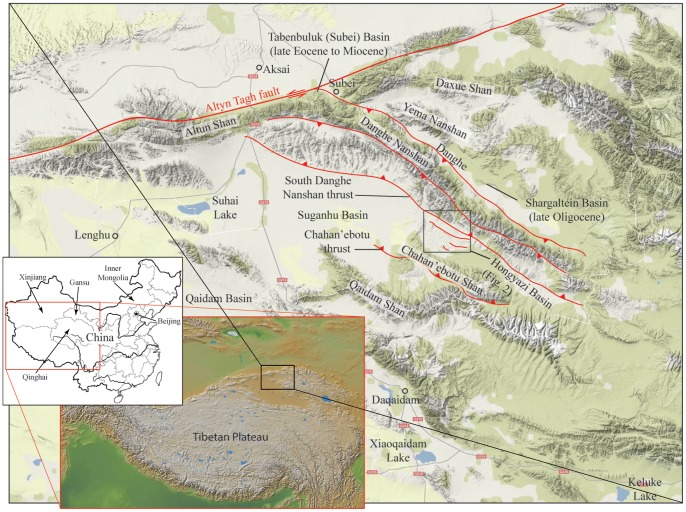
Terrain map of northern part of Qaidam Basin and surrounding mountains. Major thrust fault structures that control the development of Hongyazi Basin (small rectangle in Fig. 2) and the surrounding mountains to the north and south after Yin et al. [13]. Major Chinese western provinces that have been discussed in this paper (dotted lines within outline map of China) are indicated.

**Figure 2:**
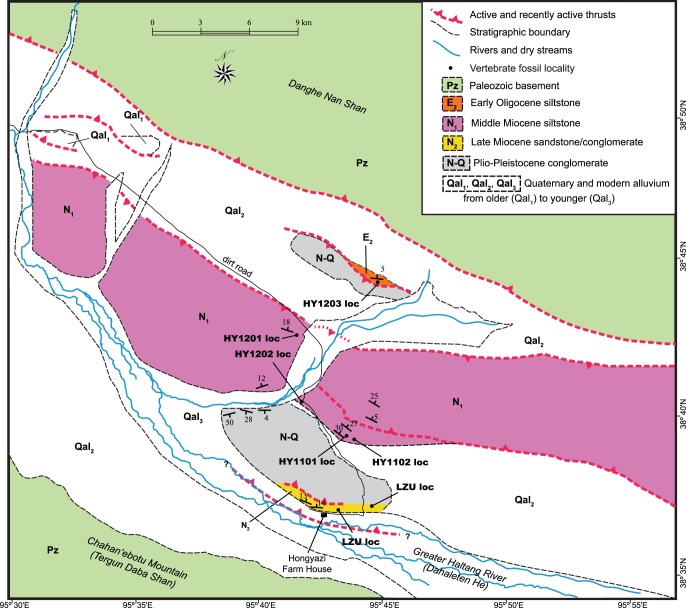
Geologic map of late Cenozoic exposures in Hongyazi Basin. See Fig. 1 for location of Hongyazi Basin. Stratigraphic boundaries, basement rocks, and faulting structures are based on a combination of our observations, Google Earth (Version 6.1.0.5001) [46], the Global Multi-Resolution Topography (GMRT) synthesis [47], GeoMapApp [48], and other sources [13], [24]. Location of the Lanzhou University fossil sites (LZU) is based on Regional Geological Survey of Gansu Bureau of Geology [24].

Tectonically, Hongyazi Basin is within the Qilian Shan-Nanshan thrust belt [13]. At a present elevation of 3,600–3,900 m above sea level, it is an intermountain basin flanked by the Danghe Nanshan to the north and Chahan’ebotu Mountain (or Tergun Daba Shan) to the southwest and drained by the Greater Haltang River (or Dahaleteng, in contrast to the Lesser Haltang River to the south of the Chahan’ebotu Mountain) (Figs. 1–2). In a NW-SE orientation, the basin is approximately 100 km long and 25 km wide in maximum dimensions. Modern drainage system is represented by a series of NE-SW braided streams, mainly sourced from snow-melts in glacial peaks in Danghe Nanshan and Chahan’ebotu Mountain that drain into the Greater Haltang River. The basin floor has an appreciable tilt toward the south and the main Haltang River is shifted southward to the northern foothill of the Chahan’ebotu Mountain.

The Greater Haltang River, often spread in a 2-km wide channel system, discharges toward the northwest and sharply bends southwestward when it clears a narrowing of the basin floor caused by a northern spur of the Chahan’ebotu Mountain (Fig. 1). Modern Hongyazi Basin is connected to the Suganhu Basin through the Greater Haltang River [14]. The timing of such a pattern of westward discharge for the Greater Haltang River is not clear.

Main axis of the regional mountains parallels those of the compressive structural regimes predominant in the northern Qaidam Basin, i.e., along a series of NW-SE fold axes and strikes of reverse faults. Along the northern flank of Hongyazi Basin, the Danghe Nanshan plays a major role in our understanding of the shortening and lateral extrusion of the northern Tibetan Plateau by frontal thrust faults and left-lateral Altyn Tagh Fault [9], [15], [16], [17], [18], [19], [20]. Along the western segment of the Danghe Nanshan near Tabenbuluk (also known as Subei or Danghe) area, a long sequence of fluvial-lacustrine and overbank deposits, up to several km thick, accumulated at the northern foothill that ranges from late Eocene through much of the Miocene [9], [21]. Toward the eastern segment of the Danghe Nanshan, sediments in Shargaltein Basin (also known as Shargaltein-Tal, near Yanchiwan; Fig. 1) are known to produce late Oligocene (Tabenbulukian) mammals [22], [23].

In Hongyazi area, along the southwestern foothill of Danghe Nanshan, a similar style of reversed fault system is the main structural element that controls the Danghe Nanshan uplift and basin sedimentation [13]. We term this thrust system South Danghe Nanshan thrust (Fig. 1), which locally cuts through sediments of early Oligocene through late Miocene age. We did not observe the sediment contact with basement rock, and the start of basin sedimentation may be slightly earlier than early Oligocene, roughly comparable to the age in Shargaltein Basin on the northeast face of Danghe Nanshan (Fig. 1). Along the northern foothills of the Chahan’ebotu Mountain, which flanks the southern margin of the Hongyazi Basin, there is no sign of faulting. Instead, the Chahan’ebotu Mountain is structurally controlled by a thrust fault along its south margin (the Chahan’ebotu thrust; Fig. 1).

### Stratigraphy

Our analysis of the stratigraphic relationship and mammalian biochronology suggests that a series of at least five reverse faults have thrust upward, tilted, and folded Oligo-Miocene and later (Plio-Pleistocene) sediments, and exposed them on the surface. Evidences for these faults to be reversed include contact relationships of strata and drag folds.

**Figure 3:**
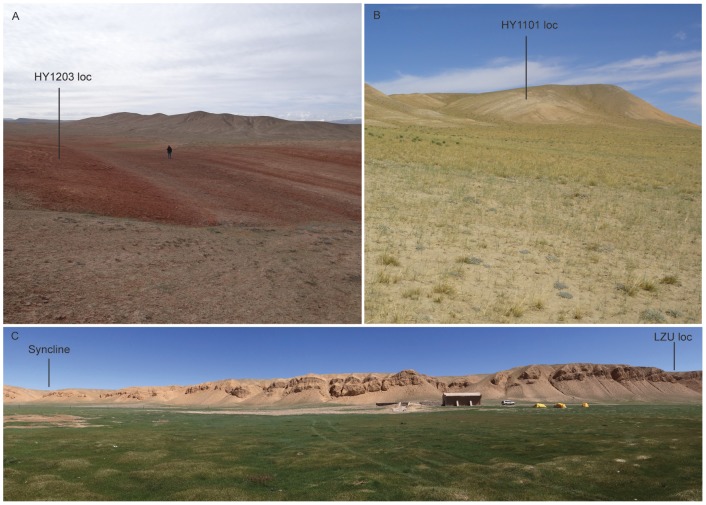
A, red mudstones and paleosols at early Oligocene HY1203 locality at the axis of an anticline; picture looking to east and person in middle is one of the authors (GX, who has given written informed consent, as outlined in the PLOS consent form, to publication of his photograph) to serve as a scale. **B**, alternating greenish and reddish siltstones at Middle Miocene HY1101 loc, photo looking to northeast. **C**, composite panorama of escarpments at Hongyazi farm house (photo looing to north), showing approximate location of late Miocene LZU loc to the east and a gentle syncline to the west. See Fig. 2 for location of above photos.

The Second Team of the Gansu Geologic Survey [24] formally named the Hongyazi Formation when it mapped Hongyazi Basin and surrounding areas, which was adopted by Gu et al. [12] and Zhang and Xie [11]. Two sections were measured and described by the survey team, both being along the main Hongyazi exposure at northern bank of the Greater Haltang River. The “East Section”, 835.4 m [11], [12] or 821 m [24], is along a canyon exposure east of the dirt road near the eastern end of the Hongyazi exposures, whereas the “West Section”, only 66.2 m in thickness, is to the west of the dirt road (solid circles labeled “LZU loc” in Fig. 2 and Fig. 3C).

The Hongyazi Formation is a sequence of yellowish to buff conglomerates, sandstones, and siltstones, the former being the dominant component, and fossil mammals were apparently recovered from a reddish brown mudstone lens in the upper part of the section [11], [12].

Vertebrate fossils are extremely rare in the very coarse-grained sediments and we were unable to relocate the fossil-producing lenses, which were presumably exhausted by the geologic survey team. Exposures along the south-facing escarpment at Hongyazi dip toward the north and form a gentle syncline with its axis roughly in north-south direction at N38°37′03″ E95°42′43″ (Fig. 3C). Because of this syncline, the Lanzhou University fossil locality (“LZU loc” in Fig. 2) in the eastern section is likely stratigraphically lower than that in the western section. Since the original localities were not relocated, we were unable to provide an exact measure of the stratigraphic range of the vertebrate fossils produced. Published faunal compositions [11], [12], however, do not indicate much time lapse between these two sites.

The Survey team has mapped all of the southern strata along the Hongyazi escarpment as Pliocene Hongyazi Formation. We, however, distinguish a dark grey to light yellow conglomerate unit either in unconformable contact on top of the Hongyazi Formation (contact relationship best seen at N38°37′04″ E95°43′22″) or cropping up as an anticline best exposed along a cliff cut by a dry wash 8 km west of Hongyazi farm house (measured section between N38°40′14.5″ E95°38′51″ and N38°40′12.6″ E95°40′04″). At this section, the southwestern limb of the anticline is more steeply dipping, up to 50 degrees, than the north limb, which eventually flattens out to be essentially flat-lying near the northeastern end of the section. The conglomerates are also seen to laterally transition into finer-grained overbank deposits, which delineate the bank of the paleochannel. We measured 147 m for the conglomerate unit. No fossil was found in this conglomerate or its finer-grained lateral extension, but its lithology somewhat resembles that of nearby dry wash beds. Contact relationship of this conglomerate (above the Hongyazi Formation) suggests that it is Plio-Pleistocene in age.

Approximately 5 km north of the Hongyazi escarpment, a long sequence of predominantly red siltstones is exposed (Fig. 3B). The Gansu Geologic Survey team used the term Baiyanghe Formation (or N_1b_ in map labels) to designate many Cenozoic red beds on either side of the Danghe Nanshan-Qilian Shan range. The type section of the Baiyanghe Formation was designated by Sun [25] in the Yümen area some 200 km northeast of Hongyazi on the opposite side of the north Tibetan range front (Danghe Nanshan – Qilian Shan). Lacking vertebrate fossils at the time of mapping, the Survey team simply referred the red beds as Miocene (or early Neogene). New fossil mammals now confirm a Miocene age for these beds in general, but our own stratigraphic boundaries differ from theirs considerably.

The Middle Miocene red bed is the largest exposed unit in the basin, spanning much of the east-west extent of the basin and the middle part of the north-south extent (Fig. 2). Most of the exposures show a monocline dipping toward the northeast, except at its southern edge, bounded by a thrust fault, where drag folding is visible (near HY1202 loc). Fine-grained, reddish mudstones and siltstones predominate in this unit with occasional thin conglomerates.

We measured a partial section in the Middle Miocene red beds for 350 m (from the lower-most exposure at HY1102 and ending at the top of a prominent hill at N38°40′32″ E95°44′50″), and there are considerably more exposed beds north of our measured section. Most of this thick sequence is difficult to correlate, except lateral tracing when well exposed, and in rare instances where a marker bed exists to aid correlation (e.g., a thin layer of dark conglomerate at top of a small, elongated hill can be matched with those on opposite sides of a big wash at N38°40′53″ E95°40′33″). So far, vertebrate fossils are only found at the southern edge (stratigraphically lower-most) of this unit (HY1101, 1102, 1202), although exposures are more numerous toward the north. When more and better fossils are discovered, further division of this unit may be warranted.

The early Oligocene red bed is only exposed at the northern end of the basin, with a limited lateral extent. This bright red (more colorful than the middle Miocene unit) mudstone has abundant carbonate nodules and gypsum crystals embedded in a paleosol. Our sole vertebrate fossil locality (HY1203) is at the axis of a small anticline. We measured 64 m for the exposed red bed and the fossil site is on top of this section. At N38°44′06″ E95°44′40″, a prominent dark grey conglomerate is in fault contact on top of the early Oligocene red bed. This conglomerate is similar in lithology to those seen further south and measured 288 m in total thickness, possibly representing a braided stream channel, and lacking any fossil evidence, we tentatively correlate it with the Plio-Pleistocene coarse-grained beds to the south.

### Vertebrate Assemblages

Overall, vertebrate fossils are still relatively rare, particularly for large mammals. However, enough fossils, especially those of small mammals, have been recovered that a broad picture in the age representation of the local strata is emerging. Three distinct faunas from three distinct stratigraphic positions and lithologies can be easily recognized. These are the Late Miocene Hongyazi Fauna from two LZU localities, Middle Miocene Ebotu Fauna from HY1101, 1102, and 1202 localities, and early Oligocene Haltang Fauna of HY1203 locality. The following are identifications of the small mammal materials recovered by us, followed by a brief note on age and faunal affinities.

#### Fossil assemblage from HY1101 locality

Erinaceinae indet. (IVPP V 18859) A single m3 confirms the presence of a hedgehog. Its size and dental morphology is consistent with ?*Mioechinus gobiensis* from the middle Miocene Tunggur Formation in Inner Mongolia. However, for lack of key molars, its identity cannot be further determined.

*Yanshuella* sp. (IVPP V 18860.1-4) Four M3 s are present. They have weak protoconules and slightly separate mesostyles. In overall shape and size, they are very similar to M3 of *Yanshuella* sp. from Tunggur.

*Desmanella storchi* Qiu, 1996 [26] (IVPP V 18861.1-8). Eight specimens have been collected, including two P4 s, two M1 s, two M2 s, one lower jaw with p4-m3, and one m1. The teeth are highly consistent with *D. storchi* from Tunggur in size and shape. M1 protoconule is large, and its metaconule is strongly extended posteriorly. The mesostyles in M1 and M2 are not separate. The p4 is simple. The crista obliqua in m1–3 are elevated to the same height as metaconids. The m3 is relatively unreduced.

Soricidae indet. (IVPP V 18862.1-8) Eight specimens have been collected, including two upper incisors, one M1, three fragmentary jaws with m1–2, m1, and m3, and an isolated m3. Very light pigmentation is still visible. Upper incisors are not bifurcated. Lengths of trigonid and talonid are roughly equal for m1–2. The talonid of m3, however, is reduced. For lack of more diagnostic materials, we cannot further identify this taxon.

**Figure 4:**
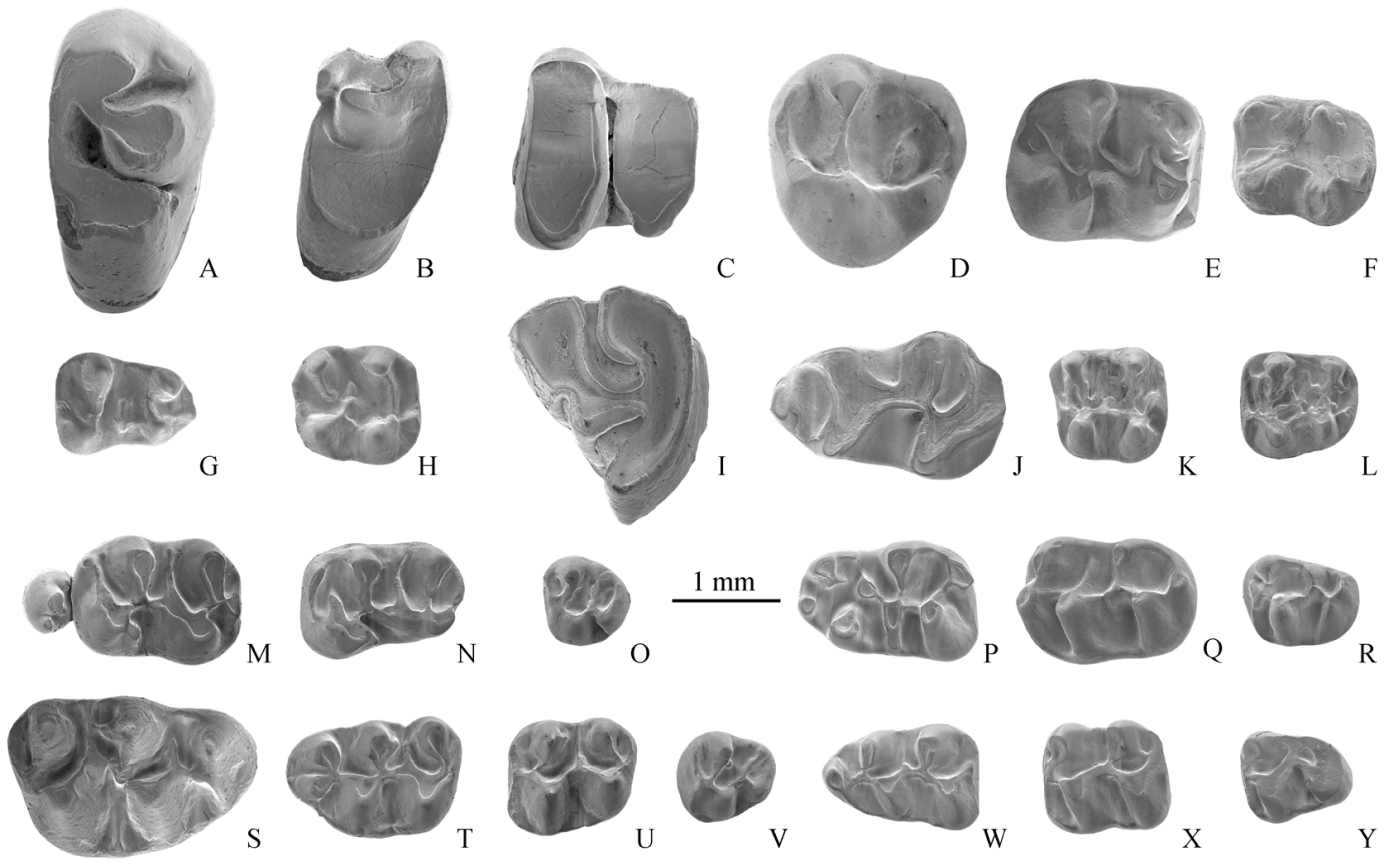
Representative small mammals from Hongyazi area, western Gansu Province. **A–H** from HY1203. **A–C**, *Desmatolagus* sp.: **A**, P2 (IVPP V 18886.1), **B**, dP3 (V 18886.4), and **C**, lower middle cheek tooth (V 18886.5). **D**, Sciuridae indet.3, M3 (V 18882). **E**, *Karakoromys decessus*, m1 or m2 (V 18883). **F**, *Karakoromys* sp., m3 (V 18884). **G–H**, Sicistini indet.: **G**, m1 (V 18885.4) and **H**, M1 (V 18885.1). **I–Y** from HY 1101.**I**, *Alloptox gobiensis*, p3 (V 18868.54); **J**, *Sayimys* sp., dp4 (V 18863). **K–L**, *Sicista* sp.: **K**, M1 (V 18864.1) and **L**, M2 (V 18864.2); **M–R**, *Heterosminthus orientalis*: **M**, P4-M1 (V 18865.2), **N**, M2 (V 18865.18), **O**, M3 (V 18865.30), **P**, m1 (V 18865.43), **Q**, m2 (V 18865.47), and **R**, m3 (V 18865.56).**S**, *Democricetodon lindsayi*, M1 (V 18867.1). **T–Y**, *Megacricetodon sinensis*: **T**, M1 (V 18866.12), **U**, M2 (V 18866.47), **V**, M3 (V 18866.72), **W**, m1 (V 18866.89), **X**, m2 (V 18866.128), and **Y**, m3 (V 18866.152). All at same scale.

*Sayimys* sp. (IVPP V 18863) Only a dp4 (Fig. 4J) is available, which is enough to show its presence in the fauna.

*Sicista* sp. (IVPP V 18864.1-2) Two specimens, an M1 and an M2, are collected. They are small in size with complex crown morphology; many secondary ridges and spurs are present; protoloph on M1 and M2 are bifurcated (Fig. 4K, 4L).

*Heterosminthus orientalis* Schaub, 1930 (IVPP V 18865.1-64). 64 specimens are recovered, including two upper jaw fragments with P4 and P4-M1, two upper jaw fragments with M1 s, one lower jaw fragment with m2–3, two lower jaw fragments with m2 s, and 57 isolated teeth (one P4, five M1 s, thirteen M2 s, eight M3 s, ten m1 s, nine m2 s, eleven m3 s). Size and crown morphology are highly consistent with *Heterosminthus orientalis* from Tunggur. The M1 and M2 mesocones are weak, with incipient development of anterostyle but no posterostyle; all have strong mesolophs. Lower molars lack stylid and ectocingulid (Fig. 4M–R).

*Megacricetodon sinensis* Qiu, 1996 (IVPP V 18866.1-159). 159 specimens have been collected, including two upper jaw fragments with M1–2, 34 M1 s, 34 M2 s, six M3 s, 38 m1 s, 32 m2 s, and 13 m3 s. Dental dimensions fall within *M. sinensis* from Tunggur Formation. Morphologically, the Hongyazi materials are also consistent with those from Tunggur, such as mesolophs in upper molars and mesolophids in lower molars having different lengths, anterocones being prominently bifurcate and different in size. M1 and M2 paracones have poorly developed ectoloph. The anteroconid in m1 has a single cusp, which is sharp and narrow (Fig. 4T–Y).

*Democricetodon lindsayi* Qiu, 1996 (IVPP V 18867.1-9). Nine specimens are available, including three M1 s, two M2 s, one M3, one m1, one m2, and one m3. Size is relatively large but falls within the range of *D. lindsayi* from the middle Miocene Tunggur Formation of Inner Mongolia. Dental morphology is also highly consistent with the latter, such as mesolophs and mesolophids in upper and lower molars being relatively long, M1 anterocone wide and single cuspid with labial spur of anterolophule (two of the three teeth have a protoloph I; Fig. 4S), and M2 metaloph forward oriented.

*Alloptox gobiensis* (IVPP V 18868.1-65). 65 specimens are recovered, including two I2 s, ten P2 s, three dP3 s, ten P3 s, 25 middle upper cheek teeth, two dp3 s, eight p3 s, two dp4 s, three middle lower cheek teeth. Size is relatively large and teeth are high crowned. P2 has only two reentrants, and p3 anterior lobe is triangular in outline without anterior reentrant (AR) (Fig. 4I). Both size and morphology of Hongyazi sample are consistent with those of *A. gobiensis* from Tunggur Formation.

**Figure 5:**
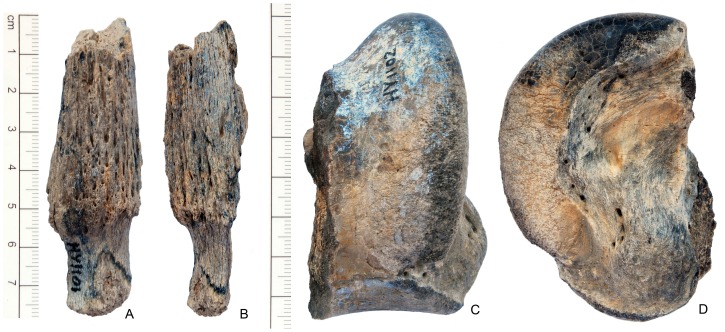
Large mammals from Hongyazi area. **A**, anterior view and **B**, lateral (or medial) view of horncore fragment of *Turcocerus* sp. (IVPP V 18869); **C**, anterior view and **D**, medial view of partial astragalus of Rhinocerotidae indet. (V 18878).

*Turcocerus* sp. (IVPP V 18869) A horncore fragment is the only specimen recovered for this taxon (Fig. 5A, B). It is a short, straight horncore, which is free of twist or a keel, and has a circular cross section, features that are typical of the primitive caprine. As pointed out by Chen [27], horncores of *T. lishanensis* have almost no twist, a character shared with the Hongyazi specimen.

#### Fossil assemblage from HY1202 locality

Erinaceinae indet. (IVPP V 18870.1-2) one P2 and one i2 are among representatives of this hedgehog.

*Microdyromys* sp. (IVPP V 18871) A single m2 indicates the presence of Gliridae. Its size and shape is close to *M. wuae* from Tunggur Formation.

*Sicista* sp. (IVPP V 18872) A single m3 is present in the sample. Secondary lophs are numerous, possibly the same taxon as that from the HY1101 locality.

Sicistini indet. (IVPP V 18873) Only an m1 is available. Very small in size, this taxon is quite different from *Sicista* above. It lacks anteroconid; its ectolophid is weak and not protruding toward the lingual side; mesolophid is very vague; secondary lophs in *Sicista* sp. are absent.

*Heterosminthus orientalis* Schaub, 1930 (IVPP V 18874.1-5). Five specimens are collected, including two M1 s, two m1 s, and one m2. Size and morphology are consistent with those of *H. orientalis* from both HY1101 locality and Tunggur Formation of Inner Mongolia.

*Megacricetodon sinensis* Qiu, 1996 (IVPP V 18875.1-5). Five specimens are available, including two M2, two m1, and one m3. Size and dental morphology is similar to those of *M. sinensis* from both HY1101 locality and Tunggur Formation.

*Democricetodon lindsayi* Qiu, 1996 (IVPP V 18876.1-5). Five specimens are collected, including one M2, one m1, one m2, and two m3 s. It is consistent with those of *D. lindsayi* from HY1101 locality and Tunggur Formation both in size and morphology.

*Alloptox gobiensis* (Young, 1932) (IVPP V 18877.1-2). Two P3 s are in the sample. Size and morphology are consistent with *A. gobiensis* from both HY1101 locality and Tunggur Formation.

Rhinocerotidae indet. (IVPP V 18878) A partial astragalus was collected (Fig. 5C, D). The size of a rhino astragalus, it has no neck between the distal articular facet and trochlea, characteristic of perissodactyls.

#### Fossil assemblage from HY1203 locality

Erinaceinae indet. (IVPP V 188791-12) Twelve fragmentary teeth are available with limited recognizable characters. The talonids of its m1 or m2 are very tall and short, and lack a cingulum at posterior rim. Instead there is a small hypoconulid. These features differ from erinaceines of early and middle Miocene. We are unable to further identify this taxon for lack of better materials.

Sciuridae indet. 1 (IVPP V 18880). One broken upper molar is available, preserving part of the crown. The tooth is large with high crown and strong ridges. Protoloph and metaloph are complete and connected to protocone. Protoconule and metaconule are somewhat swollen; mesostyle is well developed.

Sciuridae indet. 2 (IVPP V 18881.1-5). Five broken upper cheek teeth (one dP4, one P4, two M1 s or M2 s, one M3) are present. The teeth are small in size with low crown height. The protoloph and metaloph are strong and complete. No protoconule, metaconule, and mesostyle are seen.

Sciuridae indet. 3 (IVPP V 18882). A single M1 is collected. It is larger than that of Sciuridae indet. 2 above, and has a high crown and well-developed hypocone. The protoconule and metaconule are prominently enlarged. Protoloph is complete and metaloph is weak. Mesostyle is lacking (Fig. 4D).

*Karakoromys decessus* (IVPP V 18883.1-3). Only three teeth are available, including a P4, an m1, and an anteriorly broken m3. The P4 has two cusps, protocone and paracone; protoloph is complete, connecting with protocone and paracone; posterior arm of paracone converges posteriorly toward posteroloph, forming a full circle. The m1 or m2 is bunolophodont with low crowns; the lingual branch of the posterior arm of the protoconid is relatively short; trigonid basin is open; entoconid arm is transversely oriented (Fig. 4E). The above characters are similar to *K. decessus* as described by Wang [28], and its dental measurements also fall within the range of the latter.

*Karakoromys* sp. (IVPP V 18884) A single m3 (Fig. 4F) with high degree of wear is present; it is smaller than *Karakoromys decessus*.

Sicistini indet. (IVPP V 18885.1-4) Four molars (one M1 and three m1 s) are among the samples. This taxon is small in size. M1 endoloph is weak and very close to the lingual side; protoloph is not well-developed; mesocone is triangular; metaloph is transversely connected to the hypocone (Fig. 4H). Anteroconid of m1 is low; mesolophid is short; posterior part of ectolophid is undeveloped; hypolophid is transversely connected to hypoconid (Fig. 4G). This taxon may represent a new sicistine rodent pending verification by additional materials.

*Desmatolagus* sp. (IVPP V 18886.1-6) Six cheek teeth are recovered, including one P2, one dP4, one M1 or P4, one dp3, and two lower cheek teeth. Teeth are relatively small and unilaterally hypsodont. All have roots and upper cheek teeth are three-rooted. The crown of the P2 has two reentrants; middle upper cheek teeth lack lingual fold (Fig. 4A–C). Based on above characteristics, we tentatively assign this lagomorph as *Desmatolagus* sp.

### Fauna and Biochronology

#### Late miocene hongyazi fauna

A small fauna from two localities (LZU loc. in Fig. 2) within two canyons that cut into the main Hongyazi escarpment was first reported by researchers from Lanzhou University [11], [12], [29]. Of these, the eastern locality, to the east of the dirt road entering the Hongyazi escarpment, is the most fossiliferous, producing the following elements: *Hipparion platyodus*, *Hipparion* sp., *Chilotherium* cf. *C. xizangensis*, *Palaeotragus microdon*, *Gazella* cf. *G. gaudryi*, Cervidae indet., and Carnivora indet. The western locality, on the other hand, only produces *Chilotherium* cf. *C. xizangensis* and Cervidae indet. In their measured section for the eastern locality, Gu et al. [12∶80] stated that fossils came from layers 9–14 in a section of more than 835 m, although their Figure 2 placed the fossil horizon at layer 4. Zhang and Xie [11], on the other hand, more explicitly placed the fossils in layer 12. Such a discrepancy aside, it is not clear how much of the local section the two fossil localities span.

Zhang and Xie [11] proposed the name Hongyazi Fauna for this assemblage. Gu et al. [12] compared the Hongyazi Fauna to those in north China and southern Tibet. In particular, they pointed out “numerous similarities” of the Hongyazi *Chilotherium* cf. *C. xizangensis* with that from the type locality at the base of the Oma (Woma) Formation in Gyirong Basin of southern Tibet [30], which has been magnetically dated to 7.14–7.21 Ma [1], [31]. Gu et al. correlated the Hongyazi Fauna with the “Pontian age” counterparts in Europe and China, and gave it a “late Pliocene” age, as was conventional at the time. In modern terms, it is equivalent to the later part of the Miocene Baodean age [32].

#### Middle miocene ebotu fauna

Faunal assemblages from HY1101 and HY1102 localities are highly consistent with each other. They share the following common taxa: Erinaceinae indet., *Sicista* sp., *Heterosminthus orientalis*, *Megacricetodon sinensis*, *Democricetodon lindsayi*, and *Alloptox gobiensis*. Minor differences, such as soricid insectivores, *Yanshuella*, and ctenodactylid *Sayimys* so far present in HY1101 only in contrast to the presence of *Microdyromys* and a new sicistine rodent in HY1102, are likely the result of collecting biases by our limited sampling. These two localities are very close to each other geographically (Fig. 2) and stratigraphically exposed at the base of the middle Miocene siltstone sequence.

The composition of the HY1101 and HY1102 is very similar to the Moergen Fauna in the middle Miocene Tunggur Formation of Inner Mongolia [26], sharing common elements such as *Heterosminthus orientalis*, *Megacricetodon sinensis*, *Democricetodon lindsayi*, and *Alloptox gobiensis*. Furthermore, *Yanshuella* sp. and *Desmanella storchi* from HY1101 and *Microdyromys* sp. from HY1102 also have their counterparts in Tunggur. *Sayimys* is a unique ctenodactylid rodent commonly regarded as appearing in the Miocene of East or Central Asia [33]. In China, *Sayimys* is known in the late early Miocene of Sihong Fauna in Jiangsu Province [34], [35], early to middle Miocene Tiejianggou section in Tabenbuluk (Subei) Basin, Gansu Province [9], [10], [36], Guanghe area in Linxia Basin, Gansu Province [37], and Dingshanyanchi in Junggar Basin, Xinjiang [38]. Although large mammals are still very rare, presence of *Turcocerus* is consistent with a middle Miocene age. The age of the Moergen Fauna is commonly regarded as late middle Miocene, or Tunggurian land mammal age, roughly equivalent to the European Astaracian land mammal age or MN7+8 [39], [40]. The Ebotu Fauna should be in or around a similar age.

#### Early oligocene haltang fauna

So far, small mammals from the HY1203 locality are relatively few and fragmentary, and many are not easily identified to genus or species. Among the four known major groups, hedgehogs, squirrels, ctenodactylids, and sicistines, the ctenodactylid *Karakoromys decessus* is probably the most age-diagnostic. This species is only found in north China, central Mongolia, and Kazakhstan, all occurrences from early Oligocene strata [28], [41]. The morphology of Sicistini indet. is rather primitive, and is obviously different from early Miocene sicistines from Eurasia, such as *Parasminthus*, *Plesiosminthus*, *Heterosminthus*, *Bohlinosminthus*, *Litodonomys*, *Sinodonomys*, *Omoiosicista*, *Sicista*, and others. In nearby Tabenbuluk area, the early Oligocene Dingdanggou Fauna also has the *Desmatolagus*-*Karakoromys decessus* assemblage [10], [42]. Presence of these early elements clearly indicates that strata mapped as N1 by the Regional Geological Survey of Gansu Bureau of Geology [24] could not be Neogene, and may be as old as early Oligocene. Recognition of this late Paleogene fauna is important as no Paleogene vertebrate has been reported in Tibetan Plateau so far.

### Faunal Affinities

Due to high mountain barriers and unique environments, modern mammals in the Tibetan Plateau are distinct from surrounding regions, about 50% being endemic forms [43], [44]. It is thus of interest to learn the timing and detailed process of how such a unique fauna took shape. Faunal evolution in and around the plateau has been a focus in our research, both as a means to gauge zoogeographic barriers and its implication for climatic changes. A general trend of gradual faunal differentiation from surrounding regions from late Miocene onward can be recognized based on very limited information [1]. However, this picture becomes blurred toward earlier time because of our general lack of knowledge about early Miocene mammals (with the sole exception of the Xiejia Fauna in northeastern corner of Tibetan Plateau) and earlier. Our discovery of vertebrate fossils in the Hongyazi Basin is thus valuable in the availability of two new small mammal faunas from middle Miocene and early Oligocene, previously unknown anywhere within the Tibetan Plateau.

Overall both of the small mammal assemblages, Haltang Fauna and Ebotu Fauna, are essentially indistinguishable at the generic level from those elsewhere in north China, although some species level differences may exist pending additional sampling of fossil materials. The Haltang Fauna is comparable to similar early Oligocene faunas in north China, central Mongolia, and Kazakhstan. The Ebotu Fauna, on the other hand, is very similar to those in middle Miocene of Tunggur region in Inner Mongolia. The only element absent in Inner Mongolia is *Sayimys*, which is present in the early or middle Miocene of Tabenbuluk Basin. Such a similarity is particularly interesting considering that small mammals are often more regionally differentiated. For the late Miocene assemblage, the Hongyazi Fauna, it is entirely represented by large mammals, which also largely resembles *Hipparion* faunas in north China, except its chilothere rhino, which was favorably compared to those from the Gyirong Basin from southern Tibet [12]. This is in contrast to the Shengou Fauna in the nearby Qaidam Basin that features some forms only found within the Plateau, such as primitive deer, basal Tibetan antelope (*Qurliqnoria*), and musk ox *Tsaidamotherium* [1], [6], [45]. The Hongyazi Fauna has not been fully described, but based on the published faunal list it is roughly comparable in age to the Shengou Fauna, possibly slightly younger.

It thus appears that in Oligocene through late Miocene the Hongyazi area shows no sign of zoogeographic differentiation from North China and Central Asia, and the Danghe Nanshan, if already present during that time, was probably not a barrier for faunal exchanges. In contrast, during the late Miocene, the Hongyazi Fauna may have a modest differentiation from its distinctive counterpart in the Qaidam Basin, although the Hongyazi Fauna is still too poorly understood to draw a definitive conclusion.

### Tectonic Implications

The Hongyazi Basin strata are not well exposed enough to get a sense of either basement contact or syntectonic growth strata as the Danghe Nanshan was exhumed and uplifted. Based on the stratigraphic relationships and chronologic control by fossil mammals, the following can be inferred and tested by future studies.

Hongyazi Basin began to receive sedimentation in approximately early Oligocene, slightly earlier than those from Shargaltein Basin (near Yanchiwan) at the northern foothills of the Danghe Nanshan. Age controls from these two basins on either side of the eastern Danghe Nanshan thus establish initial sedimentation in this segment of the Danghe Nanshan. However, our field study has yet to reveal whether or not syntectonic growth strata are present. To the western segment of the Danghe Nanshan, at the Tabenbuluk (Subei) Basin, sedimentation began earlier in the early Eocene, again, confirmed by vertebrate fossils. Given the above differential timing of the onset of sedimentation, an eastward propagation of the Danghe Nanshan seems likely.

Sedimentation continued through middle and late Miocene, as demonstrated by vertebrate fossil records, and probably later, although no fossil was recovered from the upper conglomerates. Post-late Miocene or later thrust faults cut through the entire sedimentary package, exposing earlier strata in the north and later strata in the south, i.e., greater displacement toward the foothills of Danghe Nanshan than toward southern part of the basin.
